# Expression of Concern: Stromal interaction molecule 1 (STIM1) regulates growth, cell cycle and apoptosis of human tongue squamous carcinoma cells

**DOI:** 10.1042/BSR-20160519_EOC

**Published:** 2021-04-19

**Keywords:** apoptosis, cell cycle, STIM1, Tca-8113 cells, tongue squamous carcinoma

The Editorial Office has been made aware of potential issues surrounding the scientific validity of this paper, hence has issued an expression of concern to notify readers whilst the Editorial Office investigates.

